# Field theory for biogeography: a spatially explicit model for predicting patterns of biodiversity

**DOI:** 10.1111/j.1461-0248.2009.01404.x

**Published:** 2010-01

**Authors:** James P O'Dwyer, Jessica L Green

**Affiliations:** 1 Center for Ecology and Evolutionary Biology, University of OregonEugene, OR 97403 5289, USA; 2 Santa Fe Institute1399 Hyde Park Road, Santa Fe, NM 87501, USA

**Keywords:** Biodiversity, ecological drift, neutral theory, spatial ecology, species–area relationship, stochastic models

## Abstract

Predicting the variation of biodiversity across the surface of the Earth is a fundamental issue in ecology, and in this article we focus on one of the most widely studied spatial biodiversity patterns: the species–area relationship (SAR). The SAR is a central tool in conservation, being used to predict species loss following global climate change, and is striking in its universality throughout different geographical regions and across the tree of life. In this article we draw upon the methods of quantum field theory and the foundation of neutral community ecology to derive the first spatially explicit neutral prediction for the SAR. We find that the SAR has three phases, with a power law increase at intermediate scales, consistent with decades of documented empirical patterns. Our model also provides a building block for incorporating non-neutral biological variation, with the potential to bridge the gap between neutral and niche-based approaches to community assembly.

*Ecology Letters* (2010) 13: 87–95

## Introduction

The species–area relationship, or SAR (Preston 1960, 1962; MacArthur & Wilson 1963; May 1975; Connor & McCoy 1979; Rosenzweig 1995; Tjorve 2003), characterizes the increase in the observed number of species with increasing sample area, and has been referred to as the closest thing to a law in ecology (Lawton 1999). The SAR has played a seminal role in understanding the generation and maintenance of biodiversity, and forms a crucial basis for estimates of extinction due to habitat loss (May *et al.* 1995; Thomas *et al.* 2004). A number of different shapes have been proposed for the relationship (Rosenzweig 1995; Tjorve 2003), but one of the most generally accepted SARs falls into three distinct phases, with the different phases applying as sample area is increased from local to continental scales (Preston 1960; Williams 1964; Brown 1995; Rosenzweig 1995; Hubbell 2001). This triphasic SAR has an inverted S shape (Williams 1964), so that there is a steep increase in species at very local scales, followed by levelling off at intermediate scales and an accelerating increase in species number with area at the very largest, continental scales. The intermediate phase has commanded particular attention, and it has been proposed that over these scales species number increases as a power of area sampled, following the power law curve introduced by Arrhenius (Arrhenius 1921). This power law behaviour has been identified across a broad range of geographical regions (Rosenzweig 1995; Drakare *et al.* 2006) and across the tree of life (Green *et al.* 2004; Horner-Devine *et al.* 2004; Green & Bohannan 2006), but the reasons for the ubiquity of the power law SAR, and the forces driving the value of its exponent have yet to be determined definitively from first principles.

One of the earliest approaches to understanding the SAR was introduced by Preston (1960, 1962), who demonstrated that if the distribution of species abundances followed a lognormal distribution, then the number of species present in a random sample increases as a power law with increasing sample size, with the power law exponent close to 0.25. May later considered a wider range of possible species abundance distributions than Preston (May 1975), but found that the exponent of this power law would still be within a narrow range, typically between 0.15 and 0.4, consistent with a wide range of empirical results. The weakness of this framework is that real communities tend to exhibit spatial clustering (Plotkin *et al.* 2000b), so that individuals are more likely to be found near their conspecifics, violating the assumption that a spatial sample is equivalent to a random sample. More recent top-down approaches have made a range of different assumptions for this spatial clustering (Harte *et al.* 1999; Martin & Goldenfeld 2006; Harte *et al.* 2009), to test its impact on the SAR, and one influential example is the assumption of self-similar spatial aggregation of individuals (Harte *et al.* 1999). However, spatial clustering appears not to be self-similar with sufficient generality (Plotkin *et al.* 2000a) to provide a universal explanation for the shape of the SAR.

An alternative strategy, avoiding *a priori* assumptions for the distribution of species abundances or the spatial clustering of individuals, is to model a community from the bottom-up. This means that we specify some mechanistic rules for the behaviour of individuals, and then see what macroecological patterns emerge. An example of this approach is the neutral biodiversity theory introduced by Hubbell (2001), building on earlier work (Watterson 1974; Caswell 1976), and extensively developed (Chave & Leigh 2002; Volkov *et al.* 2003; Chave 2004; Etienne 2005; Etienne *et al.* 2007; Rosindell & Cornell 2007; Aguiar *et al.* 2009; O'Dwyer *et al.* 2009) in recent years. Neutral communities are idealized approximations where patterns are assumed to be primarily driven by the effects of stochasticity, but the present lack of a neutral prediction for the SAR reflects an outstanding mathematical problem in theoretical ecology: the combination of stochastic dynamics with a continuous spatial landscape (Durrett & Levin 1994; Bolker & Pacala 1997). Progress in dealing with stochasticity in continuous space has been limited by the lack of a practically useful, flexible mathematical framework, with the consequence that it has not so far been possible to derive a theoretical, bottom-up prediction for the SAR.

**Figure 1:**
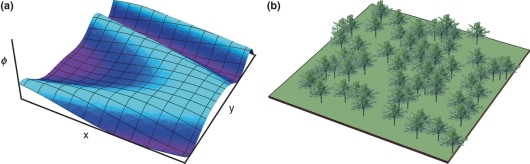
(a) Snapshot of a fluctuating field, *φ*(*x*,*y*), where for example *φ* could be the net magnetization of a ferromagnet, as a function of spatial coordinates *x* and *y*. The key object in statistical field theory is a partition function, which is defined in terms of a sum over all possible field configurations *φ*(*x*, *y*). Our model is conceptually closer to (b), with individuals of different species (a single species shown) located on a spatial landscape according to the stochastic processes of birth, death and dispersal. Some regions are densely populated, and others more sparsely, and the analogue to the field strength, *φ*(*x*, *y*) is the density of individuals as a function of spatial location.

Our goal is to overcome precisely this problem, and quantum field theory provides the perfect set of tools. Field theory was first developed as a model for particle physics (Schwinger 1958), where collisions of electrons and photons are expressed in terms of a theory of fluctuating electromagnetic fields. The same formalism has been applied to solve many-body problems in numerous fields, including the theory of phase transitions and critical phenomena, where the fields are reinterpreted as fluctuations in the density of a gas, or as fluctuations in the magnetization of a ferromagnetic material at a critical point. The central tool used to solve these problems is a moment generating functional, or partition function, which summarizes all the observable spatial patterns in these systems, and the challenge of solving a field theory is in solving for this partition function (Ryder 1996; Zinn-Justin 2002). Our key step is the introduction of a partition function for spatial ecology, illustrated conceptually in Fig. 1. Our methods follow earlier work in size-structured community assembly (O'Dwyer *et al.* 2009), and our biogeographical field theory provides a very general framework to make calculations for discrete individuals undergoing stochastic processes on a continuous spatial landscape. This flexibility also opens up the possibility for a more comprehensive understanding of spatial community assembly, with the potential to break neutrality and test which biological processes have the most impact on the macroscopic patterns we observe in nature.

In this article we begin by deriving a spatially explicit generalization of neutral biodiversity theory, on a spatially discrete, grid-like landscape, as a first step towards building a framework for spatial ecology on a continuous landscape. We then define the theory in continuous space by introducing a partition function, and we find that the partition function satisfies a functional differential equation, analogous to the Schwinger-Dyson equations of quantum field theory (Zinn-Justin 2002). Having derived the defining equation for our model, we solve for the SAR, and we also derive the expected total number of individuals as a function of area, and the turnover in species composition with spatial separation, relating these quantities to our prediction for the SAR. We conclude by discussing the significance of our results for predicting spatial patterns of biodiversity, and detail the ways in which our model can be generalized to integrate non-neutral approaches to community assembly.

## Materials and methods

### Neutral theory on the lattice

Our goal is to develop a spatial generalization of neutral biodiversity theory, a theoretical framework for community assembly introduced by Hubbell (2001). As a first step towards formulating neutral theory on a continuous landscape, we begin with the simpler case of a spatially discrete landscape. In this discrete world, individuals occupy the cells of a lattice, and the spatial location of these cells is labelled by a discrete index, *i*. The cornerstone of neutral biodiversity theory is the species abundance distribution, *P*(*n*), which is the probability that a species picked at random from a community has an abundance of *n* individuals. We now introduce the spatially explicit generalization of this distribution, which is the probability *P*(…,*n*_*i*_…,*t*) that a species has *n*_*i*_ individuals at each spatial location, *i*. This probability is conceptually similar to the species abundance distribution, only now we are taking account of the spatial location of individuals, as well as their abundance.

Individuals in our spatially discrete model die with a per capita mortality rate, *d*, and produce new offspring at a per capita birth rate, *b*, and the assumption of neutrality means that these demographic rates apply across all species. The new feature we are adding is that an individual may also be dispersed at birth to a different spatial site from its parent, thus capturing the biological process of seed dispersal by plants or trees. For an infinite landscape, *i* takes on an infinite number of values, and we can implement the dynamics of birth, death, dispersal to derive a master equation for the dynamics of *P*(…,*n*_*i*_,…,*t*) (Hubbell 2001; Volkov *et al.* 2003). If at least one *n*_*i*_ is non-zero, then the dynamics of our spatially explicit abundance distribution are described by 
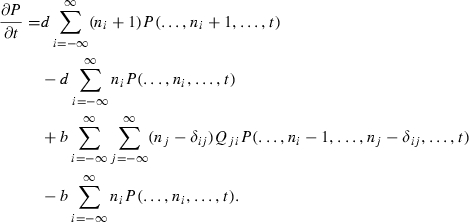
(1)

This equation generalizes the neutral theory master equation, and describes the fluctuation in abundances of individuals in space, as births, deaths and dispersal events occur through time, each pair of terms reflecting the effect of a possible transition between two different spatial configurations. The first two terms capture the effect of mortality, where the death of an individual can either add or subtract from the probability of the system being in a particular spatial configuration, and the final two terms characterize the birth process, in combination with the dispersal of seeds from site *i* to site *j*. This dispersal occurs with probability *Q*_*ij*_, and in biological terms, we would typically expect the probability of dispersal *Q*_*ij*_ to be a function of the geographical distance between sites *i* and *j*. The Kronecker symbol, *δ*_*ij*_, is zero when *i* is different from *j*, but one when *i* = *j*, accounting for dispersal from and to the same site.

We have captured the dynamics of individuals in space, but what happens when a species goes extinct, so that all values of *n*_*i*_ are equal to zero? In spatially implicit neutral models, *d* is assumed to be slightly greater than *b*, so that every species eventually dies out completely, and these extinctions must be balanced by speciation. The speciation process in neutral theory is most often modelled so that each new species begins with a single individual, and the impact of this process on the species abundance distribution is to introduce a possible transition from abundance *n* = 0 to *n* = 1. In other words, this way of modelling speciation can be thought of as a special kind of immigration event (Etienne *et al.* 2007), introducing a single individual taken from the pool of all possible species. We introduce the same process in our model, with the effect of adding two additional terms to eqn 1: 
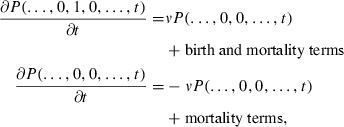
(2) where *ν* measures the rate of speciation and the effect of mortality is the same as in eqn 1.

### Neutral theory in continuous space

This discrete world represents only a rough approximation to a real community, and we now build on this discrete model to describe neutral processes on a continuous landscape. There are two key reasons for tackling this problem. First, in a real biological system individuals are not constrained to sit on a perfect grid of cells, and so by describing processes in continuous space, we better represent the processes occurring in nature. Second, the methods we introduce to combine continuous space with the stochastic processes of birth, death and dispersal naturally lead to an equation for the species-abundance distribution, a fundamental spatial biodiversity pattern, and the key prediction we would like to make using our spatial neutral theory.

However, in going beyond the discrete approximation we run up against a problem: it is not possible directly to take a continuum limit of our discrete master equation, eqn 1. Before considering continuous space, we must first rewrite the dynamics of our discrete community in terms of a moment-generating function. This generating function is defined by a sum over all spatial configurations of individuals: 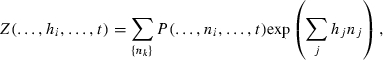
(3) and the definition means that derivatives of *Z*(…,*h*_*i*_,…,*t*) are equal to the moments of our spatially explicit probability, *P*(…,*n*_*i*_,…,*t*). Rewriting eqns 1 and 2 in terms of this generating function, we find a new master equation: 
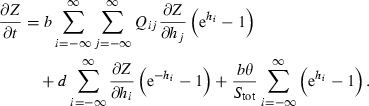
(4)

The parameters *b* and *d* are again per capita birth and mortality rates, *S*_tot_ is the total number of species across the whole landscape, and *θ* = *S*_tot_*νP*_0_/*b*, where *P*_0_ = *P*(0,0,…). The parameter *θ* is therefore precisely the neutral theory fundamental diversity parameter (Hubbell 2001; Volkov *et al.* 2003), measuring the rate per generation of new species entering the community through speciation.

It is now possible to take the limit as the spacing between discrete sites on the grid goes to zero, and in this limit the discrete set of variables *h*_*i*_, introduced in the definition of the moment-generating function, eqn 3, becomes a continuous function of spatial coordinates, *H*(*x*,*y*). Correspondingly, the generating function itself becomes a function of *H*(*x*,*y*), which we can write formally as a functional, 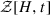. In statistical field theory 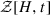 is known as a partition function, and so we use this terminology here: 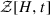 is the partition function for neutral spatial ecology. We describe the details of the continuum limit in our *Supporting Information* section, and find that the partition function satisfies the following functional differential equation: 
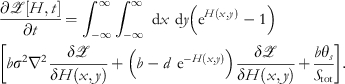
(5)

In deriving this equation, we have approximated dispersal as a diffusion process, with a length-scale *σ*, characterizing the average geographical distance traversed per dispersal event. We note that this diffusion approximation is not valid when the moments of the dispersal kernel *Q*(*x*,*y*) are not finite, as in the case of long-ranged dispersal (Clarke 1998; Clarke *et al.* 1999; Rosindell & Cornell 2009), and that the more general equations in our *Supporting Information* must be used to tackle these cases. The functional derivatives *δ*/*δH*(*x*,*y*) in eqn 5 can be thought of as a continuous space generalization of the partial derivatives, ∂/∂*h*_*i*_, in eqn 4, and similarly the sums over spatial locations in eqn 4 have been replaced by integrals over continuous spatial coordinates, *x* and *y*. The derivative operator is ∇^2^ = ∂^2^/∂*x*^2^+∂^2^/∂*y*^2^, and *θ*_*s*_ is a new fundamental measure of diversity, with dimensions *per unit area*.

## Results

### The species–area relationship

We now consider a circular sample region, of radius *R*. The equilibrium solution for the SAR is a function of *R*, and is equal to the probability of presence for species in the sampled region, summed over all extant species: 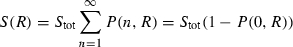
(6) where *P*(*n*,*R*) is the probability that a species picked at random from the community has *n* individuals in the sampled region, and *P*(0,*R*) is the probability that a species is completely absent from the sampled region. The second equality in eqn 6 holds because these probabilities must sum to equal 1. We can find *P*(0,*R*) from the following generating function: 
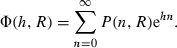
(7)

We note that from the definition of Φ(*h*,*R*), as the parameter *h* becomes large and negative, every term in this expansion is exponentially suppressed except for the lowest order term, *P*(0). In the limit of *h* → − ∞, we pick out precisely this term, and have that Φ(−∞,*R*) = *P*(0,*R*). This means that if we compute Φ(−∞,*R*), we have *P*(0, *R*), and can use eqn 6 to make a prediction for the SAR.

To derive the equilibrium solution for the SAR, we must consider equilibrium solutions for our partition function, which satisfy a local, time-independent version of eqn 5: 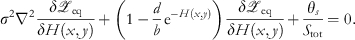
(8)

In our *Supporting Information*, we use this equation to derive an exact equilibrium solution for ∂Φ/∂*h*, in closed-form. The solution is as follows: 
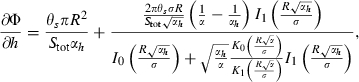
(9) where the functions *I*_0_, *I*_1_, *K*_0_ and *K*_1_ are modified Bessel functions. The parameter *α* = *d*/*b* − 1 depends on both birth and mortality rates, and the parameter *α*_*h*_ = (*d*/*b*)e^−*h*^− 1 also depends on *h*, introduced in the definition of Φ(*h*,*R*).

We still need to integrate our solution for ∂Φ/∂*h* with respect to the parameter *h*, so that we can combine eqns 6 and 7 to make our prediction for the SAR. This integration may be completed numerically, but is closely approximated by expanding the Bessel functions 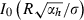 and 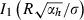 in powers of *α*_*h*_, in which case the integral can be completed analytically. Using this expansion and keeping the lowest order terms in *α*_*h*_, we find that the scaling of species number with radius of the sampled area is: 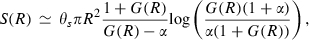
(10) where the function *G*(*R*) is a combination of Bessel functions: 
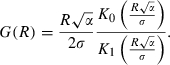
(11)

The validity of approximating the numerical integration of eqn 9 in this way is considered in more detail in our *Supporting Information*.

**Figure 2:**
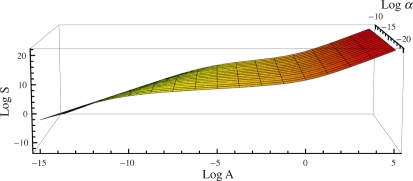
The species–area relationship (SAR) as a function of the demographic parameter *α*, and with the dimensionless combination of parameters *θπσ*^2^/*α* fixed to be 1000. On the *z*-axis is the logarithm of species number, *S*, on the *x*-axis the logarithm of sampled area, *A* and on the *y*-axis, the logarithm of *α*. Area is measured in units of *σ*^2^/*α*, so that the transition to the large-scale linear phase occurs at *A* = 1 and hence log *A* = 0, in these units. The SAR displays three distinct phases, with close to linear behaviour for small areas, exactly linear behaviour for large areas and approximately power law behaviour at intermediate scales. As *α* becomes smaller the central phase becomes broader and the exponent of the approximate power law decreases. The region shaded red indicates the large-scale linear phase, the linear shaded yellow the power law phase and the region shaded green the small-scale sampling phase.

This prediction for the SAR exhibits a classic triphasic pattern (Williams 1964; Brown 1995; Rosenzweig 1995), and is also qualitatively consistent with previous computer simulations of neutral communities (Durrett & Levin 1996; Rosindell & Cornell 2007). The parameter *α* is crucial in determining the ranges of the different phases and the steepness of the SAR in each phase, and in Fig. 2 we plot our SAR for varying values of *α*. First, for small areas, *A* < *σ*^2^, there is a steeply rising ‘sampling’ region, where the SAR is close to linear and most new individuals sampled belong to distinct species. Next, for sample areas between *σ*^2^ < *A* < *σ*^2^/*α*, there is a phase closely approximated by a power law: 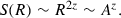
(12)

**Figure 3:**
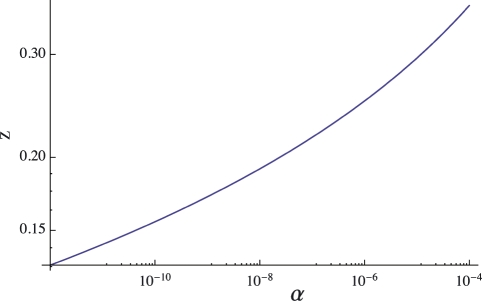
For intermediate scales our species–area relationship is closely approximated by a power law, where the exponent of the power law, *z*, depends on the demographic parameter, *α*, as shown above plotted on a log-linear scale.

While the SAR (eqn 10) is clearly not a true power law as a function of area, in this region the derivative of log *S* with respect to log *A* is very slowly varying, and so a power law is an excellent approximation. This middle region of the SAR extends over a longer range as the parameter *α* becomes smaller, and for small *α* the exponent *z* is given by: 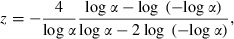
(13) where we have evaluated the slope at log *R*=log *σ*−(1/4)log *α*, in the centre of the power law region on a logarithmic scale, and we note that there is no dependence of *z* on the dispersal length-scale, *σ*. This analytical result for the exponent *z* highlights the power of our framework, and we plot the variation of *z* with varying *α* in Fig. 3. Finally, beyond the power law region, for sample areas *A* > *σ*^2^/*α*, we find a linear SAR with 
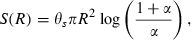
(14) so that the parameter *σ* drops out of the SAR altogether. This large-scale linear behaviour is to be expected at scales when the turnover in species composition is high.

### Applications

How do we compare our prediction for the SAR with empirical data, or use it to predict biodiversity at scales beyond which data are available? To do this we need some way to estimate the three free parameters in eqn 10. These are the fundamental diversity parameter per unit area, *θ*_*s*_, which derives from the process of speciation, the length-scale *σ*, which is the typical distance dispersed by a seed away from its parent, and the parameter *α* = (*d*/*b*) − 1, which depends on the per capita demographic rates *b* and *d*.

We can tackle this question by extracting two more equilibrium solutions from our master equation, eqn 5. First, we look for the expectation value of abundance per unit area, summed over all species, 〈*J*〉. In our *Supporting Information* we show that this expectation value is given by 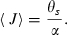
(15)

This result has a precise analogue in previous, non-spatial formulations of neutral theory (Volkov *et al.* 2003), where the expectation value for the total abundance of individuals in the metacommunity *J*_M_ = (*θ*/*α*), but in this case the spatial-explicit nature of our model naturally gives an abundance per unit area. This result means that we can exchange one of the free parameters *θ*_*s*_ or *α* for the average density of individuals in space, a straightforward quantity to measure empirically. Finally, to think of this equation another way, for fixed 〈*J*〉, we now have a direct connection between *α* and *θ*_*s*_, telling us that *α* is in effect a per capita speciation rate (Durrett & Levin 1996; Chave & Leigh 2002).

Next, we step beyond the expectation value of abundance, and look at the turnover in species composition across space, known as *β*-diversity. Using our model we derive the expectation value for *F*(*r*), a measure of *β*-diversity introduced by Chave & Leigh (2002). *F*(*r*) is the probability that two individuals picked at random, but separated by distance *r*, belong to the same species. In the *Supporting Information* we show that it has the following shape: 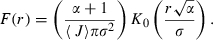
(16)*K*_0_ is a modified Bessel function, and decreases monotonically with increasing spatial separation, as we would expect for species turnover. For separations 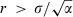, *F*(*r*) drops off exponentially quickly, so that the probability of finding two individuals of the same species becomes negligible beyond this scale. This observation adds mathematical precision to the biological intuition that species turnover should be very high at large scales, and explains why our SAR is linear for areas *A* > *σ*^2^/*α*.

Our prediction for *F*(*r*) has an identical functional form to the neutral result derived in Chave & Leigh (2002), and subsequently tested against tropical forest data (Condit *et al.* 2002). For example, fitting the per capita speciation rate, *α*, to Panamanian forest data yielded a best fit of *α* ≃ 10^−7^, and using our prediction for the SAR power law exponent, eqn 13, this value of *α* corresponds to a realistic exponent of *z* = 0.21. Our framework therefore provides a connection between the parameters underlying *F*(*r*), and the shape of the SAR, which means we can use our model to estimate total diversity at scales where comprehensive sampling of all species or taxa would be impossible. Given in particular the difficulties inherent in fully sampling microbial diversity, our framework provides a practical method for estimating taxa–area relationships (Green *et al.* 2004; Horner-Devine *et al.* 2004; Green & Bohannan 2006), under the assumption of neutral community assembly.

## Discussion

In this article we have presented a new, spatially explicit, neutral model of community assembly, and solved this model to predict a tri-phasic SAR. Our model generalizes and unifies three distinct approaches to neutral community assembly. First, we have added the biologically crucial process of dispersal to the spatially implicit neutral metacommunity introduced by Hubbell (Hubbell 2001; Volkov *et al.* 2003). Second, our framework also generalizes a previous spatial neutral prediction (Chave & Leigh 2002; Condit *et al.* 2002) for beta-diversity, *F*(*r*), for which we derive an identical functional form, and which we connect directly to the parameters underlying the shape of the SAR. Finally, our model is qualitatively consistent with computer simulations of neutral communities on a spatial grid (Durrett & Levin 1996; Rosindell & Cornell 2007; Aguiar *et al.* 2009), identifying the same three-phase SAR, and a power law exponent *z* very slowly increasing with the demographic parameter *α*. Our analytical framework goes beyond these simulated results, and allows us to make predictions in the biologically relevant limit of small speciation rate, where even the highly efficient coalescence-based methods (Rosindell & Cornell 2007) become intractable.

The three-phase SAR predicted by our model has been identified across decades of empirical studies (Preston 1960; Williams 1964; Brown 1995; Rosenzweig 1995), but it has often been thought that the pattern must arise from the effects of spatial heterogeneity: as one samples increasingly large regions, more environmental niches are uncovered, allowing increasing numbers of species to occupy these niches. Our prediction demonstrates that neutral processes and dispersal limitation alone give rise to an extremely realistic prediction for the SAR, without invoking spatial heterogeneity and environmental selection, and shows that a power law SAR at intermediate scales arises naturally from the combination of speciation with local dispersal. Of course, this does not rule out environmental heterogeneity as an important, or even the primary driver of the SAR, and to compare the effects of dispersal and heterogeneity quantitatively we will need to extend our framework to integrate both. But our results demonstrate that dispersal limitation certainly can play an important role in determining spatial structure in ecological communities.

We have shown that the exponent of the power law phase of the SAR can be expressed directly in terms of the demographic parameter, *α*, which is in turn related to speciation rate through eqn 15. We find that the exponent, *z*, increases extremely slowly for increasing speciation rate, and that for biologically realistic values of *α* taken from tropical forests (Condit *et al.* 2002), *z* is in the canonical range. Our prediction for *z* increasing with speciation rate invites comparison with empirical data. Across different geographical locations there is evidence that both power law exponent (Drakare *et al.* 2006) and speciation rate (Allen & Gillooly 2006) increase with decreasing latitude, consistent with our results. Neutral biodiversity theory has also begun to be tested across the tree of life (Sloan *et al.* 2006), and so it is natural to ask whether the relatively low reported values of *z* reported for microbial taxa–area relationships (Green *et al.* 2004; Horner-Devine *et al.* 2004; Green & Bohannan 2006) are also consistent with our model. The low values of *z* may be due to undersampling (Woodcock *et al.* 2006), or to subtleties in finding the appropriate definition of taxa (Horner-Devine *et al.* 2004), but could also indicate that microbial life has a relatively low rate of diversification. Whether microbial speciation rates are high or low has been argued in both directions, but it has been hypothesized that lower speciation rates are to be expected (Green & Bohannan 2006), consistent with a correspondingly low value of the exponent, *z*.

There are a number of possible extensions of our model. First, we have made the simple choice of a circular sample area, and exploring different geometries of sample area represents an important extension of our results. We expect that qualitatively different shapes of sample area will give quite different SARs (Kunin 1997), and so characterizing the dependence of the observed species on both area and geometry may have important applications in species conservation. Second, in deriving our central equation, eqn 5 we approximated dispersal as a diffusion process. Long-ranged dispersal occurs in many ecological communities (Clarke 1998; Clarke *et al.* 1999; Rosindell & Cornell 2009), and so developing a solution for the SAR beyond the diffusion approximation is likely to be crucial in comparing the results of our framework with empirical data. Finally, while the validity of the neutral approximation has been discussed at length (Hubbell 2001; Chave *et al.* 2002; Chave 2004; Alonso *et al.* 2006), our framework has the potential to take the debate forward quantitatively. There are several ways to break neutrality in our framework, and our approach offers the potential to derive analytical results for the relative impact of demographic stochasticity in comparison with other forces driving spatial patterns of biodiversity. For example, we could introduce a range of different dispersal capabilities for different species, or allow for spatial heterogeneity, so that a given species fares better or worse in different spatial locations (Pickett & Cadenasso 1995; Rosenzweig 1995). But a third important way to break neutrality is the introduction of biologically realistic interactions between individuals, for example the density dependence arising from competition for resources. Interactions in our model would naturally be represented by higher-order functional derivatives in eqn 5, and terms of this type have precisely the same form as interaction terms in a quantum field theory (Zinn-Justin 2002).

Our model is the first theory of spatially explicit community assembly which allows for the analytical derivation of the SAR, and in particular for the exponent of the power law phase. It is also the simplest application of a very general toolbox, which introduces the methods of field theory to biogeography, and allows us to overcome the problems of combining demographic stochasticity and a continuous spatial landscape. Our theory of individuals undergoing stochastic birth, death and dispersal certainly does differ from a typical quantum field theory, and the equations we derive are different from the field theories used to describe particle physics or critical phenomena. But the language of the partition function is universal, and the access to the tools of field theory opens up the opportunity to develop a much more general understanding of spatial patterns in ecology.

## SUPPORTING INFORMATION

Appendix S1 contains detailed descriptions of the continuum limit, the derivation of the Species-Area relationship and the derivation of spatial correlation functions.

As a service to our authors and readers, this journal provides supporting information supplied by the authors. Such materials are peer-reviewed and may be re-organized for online delivery, but are not copy-edited or typeset. Technical support issues arising from supporting information (other than missing files) should be addressed to the authors.
