# Supplementary material for: Field theory for biogeography: a spatially explicit model for predicting patterns of biodiversity
Source: Ecol Lett. 2010 Jan;13(1):87–95. doi: 10.1111/j.1461-0248.2009.01404.x (PMC2810436; doi:10.1111/j.1461-0248.2009.01404.x)
Supplement: Supplementary file 1 [file ele0013-0087-SD1.pdf]

Supporting Information

J.P O'Dwyer[1] & J.L. Green[1,2]

[1]Center for Ecology and Evolutionary Biology, University of Oregon, Eugene, OR 97403-5289
[2]Santa Fe Institute,1399 Hyde Park Road, Santa Fe, NM 87501

1 The Continuum Limit

To obtain our continuous space partition function, we multiply our discrete master equation, Eq. 1 in the main text, by the factor $e^{\sum_i h_i n_i}$, and sum over all possible spatial configurations, each spatial configuration being given by a distinct set of abundances, $\{n_i\}$. From this we obtain the following equation for $Z(\ldots, h_i, \ldots, t)$:

$$\frac{\partial Z}{\partial t} = b \sum_{i=-\infty}^{\infty} \sum_{j=-\infty}^{\infty} Q_{ij} \frac{\partial Z}{\partial h_j} \left(e^{h_i} - 1\right) + d \sum_{i=-\infty}^{\infty} \frac{\partial Z}{\partial h_i} \left(e^{-h_i} - 1\right) + \frac{b\theta}{S_{\text{tot}}} \sum_{i=-\infty}^{\infty} \left(e^{h_i} - 1\right). \tag{1}$$

This transformation means we can now take the limit as the separation between discrete spatial sites, Δx and Δy in the x and y directions respectively, goes to zero. To take this limit, we use dimensional analysis to assign the following scalings:

$$\begin{aligned} \theta &\to \theta_s \Delta x \Delta y \\ Q_{ij} &\to Q(x_i - x_j, y_i - y_j) \Delta x \Delta y, \end{aligned}$$

so that the spatially-implicit neutral theory fundamental diversity parameter, θ, becomes a new fundamental diversity parameter per unit area. We have further replaced Q_{ij}, the probability of dispersal from site i to site j, by a continuous function, known as a dispersal kernel, also with dimensions per unit area. The continuum limit of Eq. (1) is then the following functional differential equation:

$$\begin{aligned} \frac{\partial \mathcal{Z}[H,t]}{\partial t} = \int_{-\infty}^{\infty} \int_{-\infty}^{\infty} dx dy \left(e^{H(x,y)} - 1\right) \Bigg[b \int_{-\infty}^{\infty} \int_{-\infty}^{\infty} dz dw Q(w,z) \frac{\delta \mathcal{Z}}{\delta H(x+z, y+w)} \\ - d \frac{\delta \mathcal{Z}}{\delta H(x,y)} e^{-H(x,y)} + \frac{b\theta_s}{S_{\text{tot}}} \Bigg]. \end{aligned} \tag{2}$$

The functional derivatives denoted by $\delta/\delta H(x,y)$ can be thought of as a generalization to continuous space of the partial derivatives, $\partial/\partial h_i$, and are defined so that:

$$\frac{\delta H(x', y')}{\delta H(x,y)} = \delta(x' - x)\delta(y' - y). \tag{3}$$

The first term on the RHS of Eq. (2) characterizes birth and dispersal, while the remaining terms represent mortality and speciation, respectively. The exponential terms in $H(x, y)$ differentiate this from the canonical non-interacting quantum field theory (Zinn-Justin, 2002) and arise from the discrete nature of individuals.

So far we have allowed for all possible dispersal kernels, but we now model dispersal as a diffusion process. If the moments of the dispersal kernel $Q(w, z)$ are finite, we may rewrite the dispersal term in Eq. (2) as an expansion in spatial derivatives of $\delta\mathcal{Z}/\delta H$, weighted by the moments of the dispersal kernel. To derive our central equation we keep only the first two terms in this expansion, corresponding to a diffusion approximation:

$$\int dwdz\ Q(w,z)\frac{\delta\mathcal{Z}}{\delta H(x+z,y+w)} \approx \frac{\delta\mathcal{Z}}{\delta H(x,y)} + \sigma^2\nabla^2\frac{\delta\mathcal{Z}}{\delta H(x,y)} \tag{4}$$

where $\nabla^2 = \partial/\partial x^2 + \partial/\partial y^2$, and the length-scale associated with the dispersal process is σ, equal to the square root of the variance of the underlying dispersal kernel. We expect the diffusion approximation to be valid beyond very small length-scales of order $\sim \sigma$, so long as the moments of the dispersal kernel drop off sufficiently quickly, as discussed in (Chave & Leigh, 2002). Having made this diffusion approximation, we obtain the following functional differential equation for $\mathcal{Z}[H, t]$:

$$\frac{\partial\mathcal{Z}[H,t]}{\partial t} = \int_{-\infty}^{\infty}\int_{-\infty}^{\infty} dxdy\left(e^{H(x,y)}-1\right)\left[b\sigma^2\nabla^2\frac{\delta\mathcal{Z}}{\delta H(x,y)} + \left(b - de^{-H(x,y)}\right)\frac{\delta\mathcal{Z}}{\delta H(x,y)} + \frac{b\theta_s}{S_{\rm tot}}\right], \tag{5}$$

also given in the main text.

2 Equilibrium Partition Function and SAR

An equilibrium solution for the partition function is a time-independent functional, $\mathcal{Z}_{\rm eq}[H]$, which may be defined formally as the following expectation value:

$$\mathcal{Z}_{\rm eq}[H] = \left\langle e^{\int_{-\infty}^{\infty} dxdyH(x,y)n(x,y)}\right\rangle. \tag{6}$$

$\mathcal{Z}_{\rm eq}[H]$ then satisfies a local, time-independent version of Eq. (5),

$$\sigma^2\nabla^2\frac{\delta\mathcal{Z}_{\rm eq}}{\delta H(x,y)} + \left(1 - \frac{d}{b}e^{-H(x,y)}\right)\frac{\delta\mathcal{Z}_{\rm eq}}{\delta H(x,y)} + \frac{\theta_s}{S_{\rm tot}} = 0. \tag{7}$$

We can relate this equilibrium partition function to $\Phi(h, R)$, defined by Eq. 7 in the main text, by setting the function $H(x, y)$ to be a constant h inside the circular sample region, and zero outside. Making this choice for $H(x, y)$, Eq. (7) reduces to a differential equation:

$$\sigma^2\nabla^2 f(x,y,h,R) - \alpha f(x,y,h,R) + \mathrm{Rect}(x,y,R)\left(\alpha-\alpha_h\right)f(x,y,h,R) + \frac{\theta_s}{S_{\rm tot}} = 0 \tag{8}$$

where the function $f(x, y, h, R)$ is the expectation value

$$f(x, y, h, R) = \left\langle n(x, y) e^{h \int_R n(x,y)} \right\rangle . \tag{9}$$

$\mathrm{Rect}(x, y, R)$ is a two-dimensional version of the rectangular function, so that it takes the value 1 within a circle of radius R and zero outside this radius, reflecting the extent of the sampled region. The other parameters in this equation are given by:

$$\alpha = \frac{d}{b} - 1,$$
$$\alpha_h = \frac{d}{b} e^{-h} - 1. \tag{10}$$

This equation is useful because $f(x, y, h, R)$ can be related to the generating function $\Phi(h, R)$ by integrating Eq. (9) over the sampled region:

$$\int_R dxdy f(x, y, h, R) = \frac{\partial \Phi(h, R)}{\partial h}. \tag{11}$$

so that if we integrate the appropriate solution for $f(x, y, h, R)$, we have a solution for $\partial\Phi/\partial h$ as required.

The differential equation Eq. (8) can be solved by obtaining separate solutions inside and outside the sample region, and gluing them together by requiring continuity and continuity of first derivatives; the second derivative of $f(x, y, h, R)$ is clearly discontinuous, owing to the form of Eq. (8). Given the symmetries of the problem we write f as a function of polar coordinates, r and ϕ and look for a solution independent of ϕ. Inside the sample region the solution takes the form:

$$f_{\mathrm{in}}(r, h, R) = \frac{\theta_s}{S_{\mathrm{tot}} \alpha_h} + A I_0 \left(\frac{r\sqrt{\alpha_h}}{\sigma} \right) \tag{12}$$

while outside the solution is

$$f_{\mathrm{out}}(r, h, R) = \frac{\theta_s}{S_{\mathrm{tot}} \alpha} + B K_0 \left(\frac{r\sqrt{\alpha}}{\sigma} \right). \tag{13}$$

where I_0 and K_0 are modified Bessel functions, and A and B are as yet undetermined coefficients. We fix the coefficients A and B by imposing that $f_{\mathrm{in}}(R, h, R) = f_{\mathrm{out}}(R, h, R)$, and that the first derivatives of f_{in} and f_{out} are also equal at $r = R$. The relevant result is that

$$f_{\mathrm{in}}(r, h, R) = \frac{\theta_s}{S_{\mathrm{tot}} \alpha_h} + \frac{\frac{\theta_s}{S_{\mathrm{tot}}} \left(\frac{1}{\alpha} - \frac{1}{\alpha_h} \right) I_0 \left(\frac{r\sqrt{\alpha_h}}{\sigma} \right)}{I_0 \left(\frac{R\sqrt{\alpha_h}}{\sigma} \right) + \sqrt{\frac{\alpha_h}{\alpha}} \frac{K_0\left(\frac{R\sqrt{\alpha}}{\sigma}\right)}{K_1\left(\frac{R\sqrt{\alpha}}{\sigma}\right)} I_1 \left(\frac{R\sqrt{\alpha_h}}{\sigma} \right)} \tag{14}$$

Integrating this result from $r = 0$ to $r = R$, and from $\phi = 0$ to $\phi = 2\pi$, we obtain our solution for $\partial\Phi/\partial h$,

$$\frac{\partial \Phi}{\partial h} = \frac{\theta_s \pi R^2}{S_{\mathrm{tot}} \alpha_h} + \frac{\frac{2\pi\theta_s \sigma R}{S_{\mathrm{tot}}\sqrt{\alpha_h}} \left(\frac{1}{\alpha} - \frac{1}{\alpha_h} \right) I_1 \left(\frac{R\sqrt{\alpha_h}}{\sigma} \right)}{I_0 \left(\frac{R\sqrt{\alpha_h}}{\sigma} \right) + \sqrt{\frac{\alpha_h}{\alpha}} \frac{K_0\left(\frac{R\sqrt{\alpha}}{\sigma}\right)}{K_1\left(\frac{R\sqrt{\alpha}}{\sigma}\right)} I_1 \left(\frac{R\sqrt{\alpha_h}}{\sigma} \right)}. \tag{15}$$

2.1 Taylor Series Approximation for $\partial\Phi/\partial h$

We need to integrate Eq.(15) with respect to h, from $h = 0$ to $h = -\infty$ to obtain the SAR, as explained in the main text. This integral may be completed numerically, but in order to arrive at a closed form analytical result we Taylor-expanded the Bessel functions in Eq.(15) and integrated analytically. To compare with empirical data we could simply complete the numerical integral for appropriate parameter values of α and σ, but the Taylor-series result is an excellent approximation to the numerical integration, as we show in Figure 1 for $\alpha = 1$, $\alpha = 10^{-6}$ and $\alpha = 10^{-10}$:

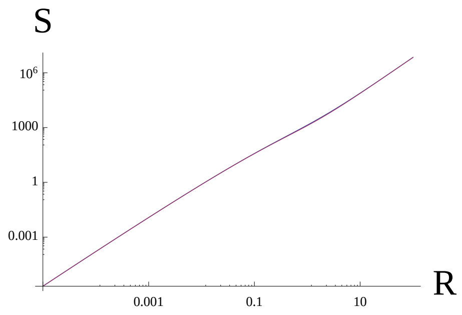

(a) $\alpha = 1$

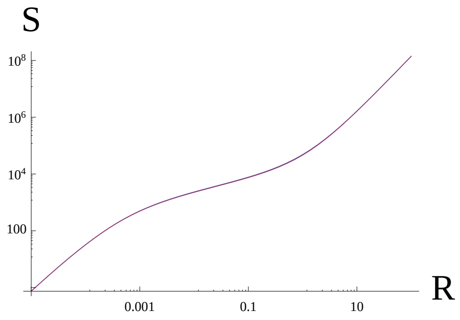

(b) $\alpha = 10^{-6}$

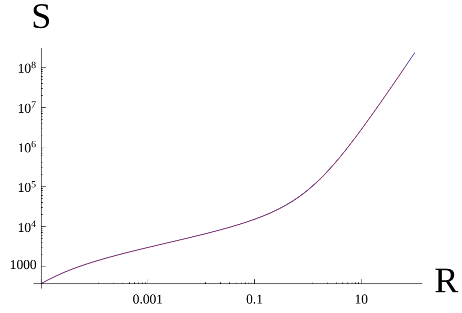

(c) $\alpha = 10^{-10}$

Figure 1: Species number S plotted against sample radius, R, with radius measured in units of $\sigma/\sqrt{\alpha}$. We plot both the numerical integration (red) and the analytical result obtained by Taylor-expanding the Bessel functions in Eq.(15) (blue). (Both lines are plotted but the two are close to indistinguishable).

3 Correlation Functions and Beta Diversity

Functional derivatives of the partition function are by definition equal to spatial correlation functions. We can therefore expand the partition function as a power series in $H(x, y)$, and extract correlation functions from the coefficients. We focus here on the first two terms in the

expansion, for the one-point and two-point correlation functions:

$$\mathcal{Z}[H,t] = \int dxdy \langle n(x,y,t)\rangle H(x,y) + \int \langle n(x_1,y_1,t)n(x_2,y_2,t)\rangle\, H(x_1,y_1)H(x_2,y_2) + \dots \tag{16}$$

Using our fundamental master equation, Eq. (5), we can derive differential equations for these correlation functions, known as Schwinger-Dyson equations in quantum field theory. To obtain these equations we insert the expansion (16) into Eq. (5), take an appropriate number of functional derivatives with respect to $H(x,y)$, and then set $H(x,y) = 0$ everywhere. For the one and two point correlation functions these equations are respectively:

$$\frac{\partial}{\partial t}\langle n(x,y,t)\rangle = \nabla^2 \langle n(x,y,t)\rangle + (b-d)\langle n(x,y,t)\rangle + \frac{b\theta_s}{S_{\text{tot}}}, \tag{17}$$

$$\begin{aligned}\frac{\partial}{\partial t}\langle n(x_1,y_1,t)n(x_2,y_2,t)\rangle &= \left(\nabla_1^2 + \nabla_2^2 + 2b - 2d\right)\langle n(x_1,y_1,t)n(x_2,y_2,t)\rangle \\ &+ \left(\nabla_1^2\langle n(x_1,t)\rangle + (b+d)\langle n(x_1,t)\rangle + \frac{b\theta_s}{S_{\text{tot}}}\right)\delta(x_1-x_2).\end{aligned} \tag{18}$$

It is similarly straightforward to derive differential equations for higher order correlation functions, by expanding the partition function to higher order in H.

Because our spatial neutral theory is non-interacting, we found only single functional derivatives in Eq. (5), and as a consequence we can solve exactly for these correlation functions, order by order. The equilibrium solution for the one-point function, which is the expectation value for the abundance of a single species, is a constant density, given by:

$$\langle n(x,y)\rangle = \frac{\theta_s}{S_{\text{tot}}}\frac{b}{d-b}. \tag{19}$$

We should expect that this expectation value is constant across space, because there are no preferred spatial locations in our spatially-homogeneous system. This result also means that the expectation value for total density of individuals, summed across all extant species, is given by

$$\langle J\rangle = S_{\text{tot}}\langle n(x,y)\rangle = \frac{\theta_s}{\alpha}, \tag{20}$$

where the dimensionless parameter $\alpha = \frac{d}{b} - 1$.

The second coefficient in the expansion of $\mathcal{Z}[H,t]$ is the two-point correlation function, $\langle n(x_1,y_1,t)n(x_2,y_2,t)\rangle$, which characterizes the spatial clustering of individuals belonging to a single species. The equilibrium solution of Eq. (18) with appropriate boundary conditions is:

$$\langle n(x_1,y_1)n(x_2,y_2)\rangle = \frac{\theta_s}{S_{\text{tot}}}\left(\frac{\alpha+1}{\pi\sigma^2\alpha}\right)K_0\left(\frac{r\sqrt{\alpha}}{\sigma}\right), \tag{21}$$

where $r = \sqrt{(x_1-x_2)^2 + (y_1-y_2)^2}$ is the separation between the two points in space and K_0 is a modified Bessel function of the second kind. The two-point function (21) characterizes the

spatial correlation of individuals belonging to a single species, but we can use our model to predict a community level measure of beta-diversity, the function $F(r)$ introduced by Chave & Leigh (Chave & Leigh, 2002). $F(r)$ is defined as the probability that two individuals picked at random from a community, but separated by distance r, belong to the same species.

To derive $F(r)$, let us first focus on one particular species, and consider the probability that two randomly-sampled individuals belong to it. This is the expectation value of the fraction of individuals belonging to this species at one spatial location, multiplied by the fraction of individuals belonging to the same species at a second spatial location. If the variation in the total density of individuals across space is negligible, this expectation value is given by:

$$\left\langle \frac{n(x_1, y_1)}{\langle J \rangle} \frac{n(x_2, y_2)}{\langle J \rangle} \right\rangle .$$

To obtain $F(r)$, we simply sum over all species, and use our assumption of neutrality. The result is

$$F(r) = \left(\frac{\alpha + 1}{\langle J \rangle \pi \sigma^2} \right) K_0 \left(\frac{r\sqrt{\alpha}}{\sigma} \right) , \tag{22}$$

which has the same functional form derived in (Chave & Leigh, 2002).

References

Chave, J. & Leigh, E. G. (2002). A spatially explicit neutral model of beta-diversity in tropical forests. *Theor Pop Biol*, 62, 153–168.

Zinn-Justin, J. (2002). *Quantum Field Theory and Critical Phenomena.* Oxford University Press, Oxford.
